# Ultrasonography and Biomarkers in the Diagnostic Evaluation of Peritoneal Tuberculosis: A Case Series Analysis

**DOI:** 10.3390/diagnostics15162008

**Published:** 2025-08-11

**Authors:** Andi Darma Putra, Gatot Purwoto, Yumeico Sachi, Chandra William Suhendar, Ilham Nugroho, Izzati Saidah, Jourdan Wirasugianto, Amelia, Lasmini Syariatin, Graciella Angelica Lukas

**Affiliations:** Division of Gynecologic Oncology, Department of Obstetrics and Gynecology, Cipto Mangunkusumo Hospital, Central Jakarta 10430, Indonesia; gatotpurwoto@gmail.com (G.P.); yumeicosch@gmail.com (Y.S.); chandrawill88@gmail.com (C.W.S.); ilhamnpriyadi@gmail.com (I.N.); izzati_saidah@yahoo.com (I.S.); jourdanwirasugianto@gmail.com (J.W.); amelsumber@gmail.com (A.); lasminisyariatin@mail.ugm.ac.id (L.S.); graciellangelica9@gmail.com (G.A.L.)

**Keywords:** ADA, biomarkers, CA-125, IGRA, peritoneal tuberculosis, ultrasonography

## Abstract

**Objectives:** This study aims to describe the ultrasound findings and biomarker profiles (CA-125, HE4, CEA, ADA, and IGRA) in confirmed cases of peritoneal tuberculosis (PTB) and to discuss their relevance in clinical evaluation. **Methods:** This is a retrospective study utilizing data from 12 female subjects with a confirmed PTB diagnosis at Cipto Mangunkusumo Hospital and Hermina Depok Hospital between 2018 and 2023. Data were extracted from medical records. Biomarker levels were measured using standardized assays in a single accredited laboratory. Ultrasonography was performed using the Mindray Resona 7 system. **Results:** The mean age was 33.0 ± 9.7 years. Ultrasonography identified significant features of PTB, such as hydrosalpinx 7 (58.3%), adhesions 6 (50%), ascites 7 (58.3%), cystic/mass-like lesions 4 (33.3%), and involvement of the rectosigmoid colon and small bowel 2 (16.6%). CA-125 levels were elevated (mean: 484.25 U/mL), and HE4 was high in 41.6% of cases (mean: 66.8 pmol/L). CEA levels remained low (mean: 1.725 ng/mL), and ADA levels were elevated in all patients (mean: 45.8 U/L). IGRA testing yielded a 75% positivity rate, with one patient converting from negative to positive after a month. **Conclusions:** Ultrasound remains a valuable imaging modality for identifying characteristic features of PTB, particularly hydrosalpinx and ascites. Elevated CA-125 and ADA, alongside IGRA results, may support clinical suspicion and help guide diagnosis in settings where invasive procedures are limited.

## 1. Introduction

**Figure 1:**
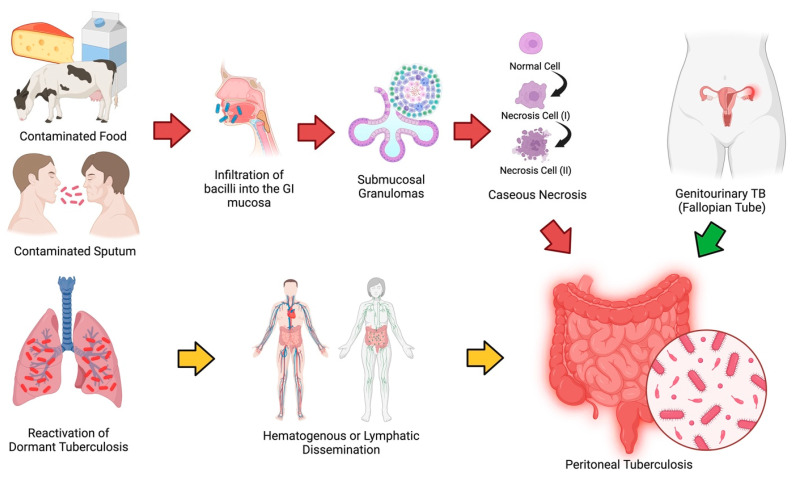
Pathogenesis of Peritoneal Tuberculosis. In the majority of cases, the bacteria propagate through the reactivation of tuberculosis in the lungs and disseminate to the peritoneum via the bloodstream or lymphatic system (yellow arrow). While less common, peritoneal infection can also occur via the gastrointestinal tract when individuals ingest contaminated sputum, unpasteurized milk, meats [5], and cheese [6]. The bacteria first infect the Peyer’s patches in the intestinal mucosa, and then spread to the mesenteric lymph nodes, where epithelioid tubercles form. Over the course of 2 to 4 weeks, caseous necrosis develops within these tubercles, leading to ulceration of the mucosal lining and potential infection of deeper intestinal layers. Eventually, the infection can spread to nearby lymph nodes and the peritoneum (red arrow). A rarer pathway of infection involves direct contamination of the peritoneum from a nearby infectious source, such as an abscess in the fallopian tubes [7] (green arrow).

Peritoneal Tuberculosis (PTB), an extrapulmonary form of tuberculosis (TB) caused by the Mycobacterium tuberculosis complex, continues to provide substantial diagnostic and treatment hurdles, eventually determining the clinical prognosis of affected individuals [1]. PTB stands as the most frequently encountered manifestation of abdominal tuberculosis, accounting for more than 15% of extrapulmonary TB diagnoses. Current estimates suggest that PTB may contribute to over two-thirds of abdominal TB cases, while less prevalent forms include intestinal, luminal, and abdominal-nodal tuberculosis [2]. PTB often results from the reactivation of dormant tuberculosis, where the bacteria spread from a previous lung infection through the bloodstream to the mesenteric lymph nodes. This leads to the activation of a localized tuberculous focus in the peritoneum. Besides hematogenous spread, PTB can also occur when the bacilli are ingested, passing through the Peyer’s patches in the intestinal lining and then to the mesenteric lymph nodes. Another route includes the spread from nearby infected lymph nodes or from ileocecal tuberculosis. In rare instances, PTB can develop through direct transmission from genitourinary sites, such as the fallopian tubes, or by the dissemination of bacteria from active pulmonary tuberculosis. While *Mycobacterium tuberculosis* is the primary cause, *Mycobacterium bovis* is a significant cause of abdominal tuberculosis, especially in areas where unpasteurized milk is consumed (Figure 1) [3,4].

Patients with PTB usually have clinical symptoms for several weeks to months before a diagnosis is obtained. The delay might present challenges, as PTB exhibits symptoms common to various other abdominal conditions. Prior research indicates that patients with PTB commonly report gastrointestinal discomfort, fever, and typical symptoms associated with tuberculosis, including weight loss, fatigue, night sweats, and anorexia [8]. Furthermore, some individuals may exhibit symptoms of pulmonary tuberculosis, such as hemoptysis. The predominant physical findings in patients with PTB include ascites and abdominal distension [9]. These clinical signs frequently overlap with other acute abdominal diseases, including ovarian cancer, thereby complicating the diagnostic process [10].

The non-specific nature of these symptoms, coupled with the limitations of diagnostic tests, renders the diagnosis of PTB challenging. The definitive method for diagnosing PTB is the detection of *Mycobacterium tuberculosis* bacteria via laparoscopic biopsy [11]. While this technique remains the most widely used, it is invasive and carries certain risks. Laparoscopic and mini-laparotomy biopsies are typically employed to obtain tissue samples for confirmation. However, these procedures are complex, require specialized clinical expertise, and may not be feasible in all healthcare settings. Ultrasonography (US) is a promising diagnostic modality that could contribute to the identification of PTB, owing to its cost-effectiveness, non-invasive nature, and widespread availability. The US has the capacity to detect peritoneal abnormalities, even in the absence of ascites, thereby providing valuable diagnostic insights. Furthermore, it can facilitate the guidance of percutaneous biopsies, offering a less invasive alternative to laparoscopy [12]. In regions with a high prevalence of PTB, certain peritoneal findings identified via US may carry significant diagnostic relevance, supporting its potential role in PTB diagnosis.

In addition to imaging, biomarkers such as CA-125 [13], human epididymis protein 4 (HE4) [14], carcinoembryonic antigen (CEA) [15], adenosine deaminase (ADA) [16], and interferon-gamma release assay (IGRA) [17] have been identified as potential adjuncts in diagnosing peritoneal TB. These biomarkers are often related to PTB patients and could serve as non-invasive indicators.

Given the diagnostic challenges associated with PTB, including its symptom overlap with other abdominal conditions and limitations of current testing methods, there is a critical need for alternative, non-invasive diagnostic strategies. This study aims to describe the ultrasound findings and biomarker profiles (CA-125, HE4, CEA, ADA, and IGRA) in confirmed cases of PTB and to discuss their relevance in clinical evaluation. By exploring these less invasive tools, the research seeks to offer practical solutions that could enhance early detection, reduce the need for invasive procedures like laparoscopy, and ultimately improve the accuracy of PTB diagnoses in clinical settings.

## 2. Materials and Methods

### 2.1. Study Design and Ethical Clearance

This is a retrospective study utilizing data from confirmed PTB patients at Cipto Mangunkusumo Hospital and Hermina Depok Hospital between January 2018 and December 2023. Data were collected through a review of medical records from the hospital databases. The data for this study were accessed in January 2024 for research purposes. During the data collection process, the authors did not have access to personally identifiable information that could link individual participants to the data. This study adhered to ethical guidelines for the use of medical records and ensured patient privacy in compliance with the Declaration of Helsinki. All patient data were anonymized before analysis to maintain confidentiality. Descriptive statistics were used to summarize the demographic characteristics, ultrasound features, and biomarker data of the study population. These data were presented in tables to highlight the distribution of key variables, including the frequency of abnormal ultrasound findings and the proportion of patients with biomarker levels exceeding normal reference ranges. The study was conducted in accordance with the Declaration of Helsinki and approved by the Ethics Committee of The Faculty of Medicine, University of Indonesia—Cipto Mangunkusumo Hospital (protocol code 24-01-0009 and date of approval 22 January 2024).

### 2.2. Inclusion and Exclusion Criteria

Inclusion criteria for this study included female patients with a confirmed diagnosis of peritoneal tuberculosis based on clinical, microbiological, or imaging findings; patients presenting with typical symptoms of PTB; and patients with available data, including demographic details, ultrasound results, and biomarker levels for CA-125, HE4, CEA, ADA, and IGRA. Only patients with sonographically confirmed ascites who had ADA measurements as part of their diagnostic workup were included for ADA analysis. Additionally, patients who provided informed consent for the use of their medical records, or for whom institutional approval was obtained for the use of de-identified data, were eligible. Exclusion criteria included female patients without a confirmed diagnosis of PTB, those with acute peritonitis or other conditions that could confuse the diagnosis, and patients without complete data. Patients with other active infections that could affect the peritoneum or interfere with the biomarkers, and pregnant women, were excluded. Lastly, patients who did not consent to the use of their medical records for research purposes were excluded.

### 2.3. Measurement of Biomarker Levels and Ultrasonography

Biomarker levels were measured in plasma (for CA-125, HE4, and CEA), blood (for IGRA), and ascitic fluid (for ADA) using standardized assays performed in a single accredited laboratory. The following values were regarded as normal blood reference levels: CA-125 ≤ 35 U/mL, HE4 ≤ 70 pmol/L, CEA ≤ 5 ng/mL, and ADA < 30 U/L. Diagnosis of PTB was primarily based on clinical evaluation, elevated biomarkers, and, in some cases, empirical treatment with anti-TB medications. In patients where the diagnosis was unclear, surgical exploration was performed, and histopathological examination of tissue samples confirmed the presence of granulomas and caseous necrosis, consistent with tuberculosis. Ultrasonography was conducted using the Mindray Resona 7 (Shenzhen, China) system with patients in a supine position. The system employed 2D Transabdominal Ultrasound (TAUS) using a 1–6 MHz abdominal transducer and Transvaginal Ultrasound (TVUS) with a 3–7 MHz endovaginal transducer. Image optimization was achieved using the time gain compensation setting. During the TAUS procedure, the transducer was placed on the lower abdomen, while the endovaginal transducer was inserted into the vagina. Images were captured in both longitudinal and transversal planes.

## 3. Results

The mean age of the 12 female patients was 33.0 ± 9.7 years, with a median of 30.5 years. Most patients were premenopausal 10 (83.3%) and married 9 (75%). A history of TB exposure was identified in 3 (25%) of cases, and histopathological confirmation of tuberculosis was obtained in 4 patients (33.3%) (Table 1).

The ultrasonography findings in this study reveal a high prevalence of chronic pelvic infections, with hydrosalpinx detected in 7 out of 12 cases (58.3%), often accompanied by adhesions and caseous degeneration, suggesting a possible tuberculous infection. Adhesion complexes were present in 6 cases (50%), affecting the fallopian tubes and ovaries. Ascites was found in 7 cases (58.3%), with some showing complex fluid or caseous degeneration. The involvement of the rectosigmoid colon and small bowel was noted in 2 cases (16.6%), indicating disease progression beyond the reproductive system. Furthermore, cystic or mass-like abnormalities in the fallopian tubes and ovaries were detected in at least 4 cases (33.3%), ranging from simple cysts to multilocular masses with complex fluid (Table 2).

**Figure 2:**
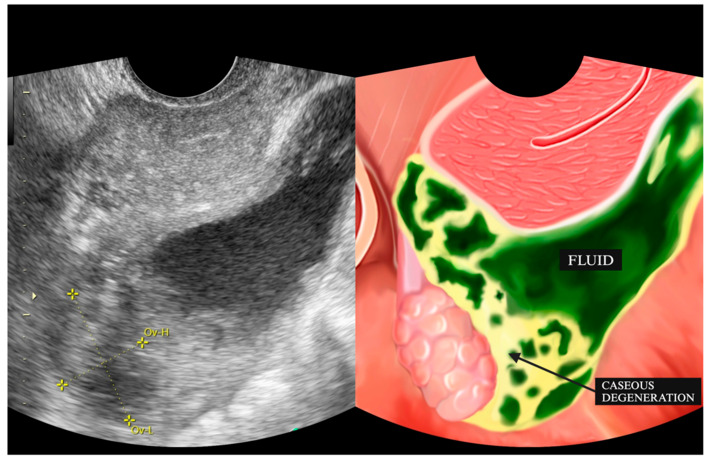
Fluid accumulation with caseous degeneration in the Douglas pouch. Ov-H: Ovary—High; Ov-L: Ovary—Low.

**Figure 3:**
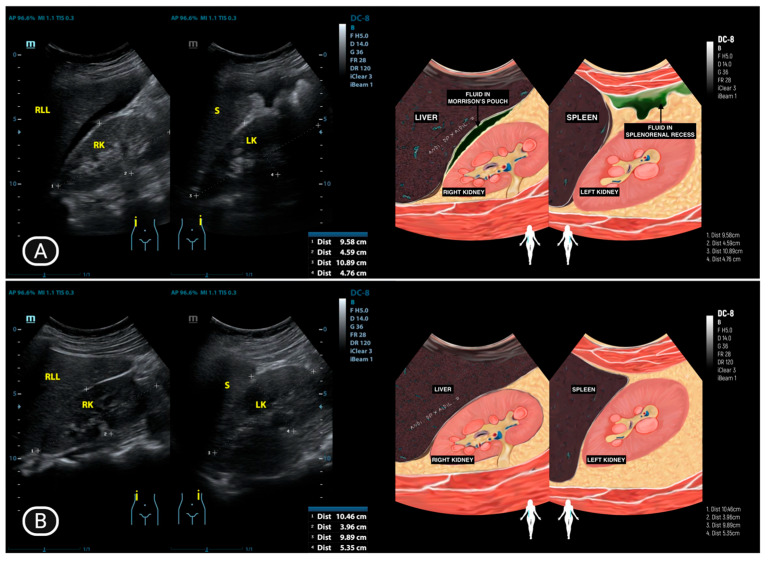
(**A**) Fluid accumulation in Morison’s pouch and splenorenal recess; (**B**) Fluid resolution after one month of anti-tuberculosis drug therapy.

**Figure 4:**
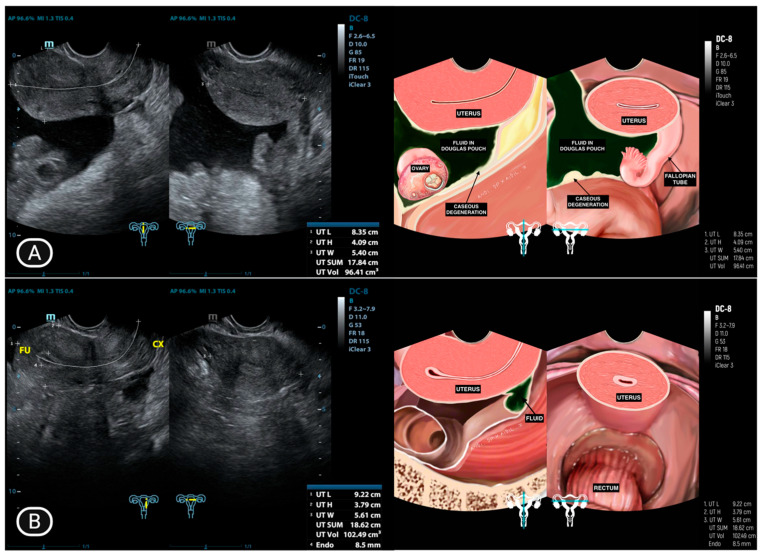
(**A**) Fluid accumulation in the Douglas pouch with caseous degeneration covering the uterus and rectosigmoid colon; (**B**) Significant reduction in fluid and caseous degeneration after one month of anti-tuberculosis drug therapy.

The presence of ascites is illustrated in Figure 2, Figure 3 and Figure 4, where it appears as an anechoic or hypoechoic space with debris or septa (complex ascites) between the organs. Figure 3B and Figure 4B provide a comparison of ultrasound images taken after one month of anti-tuberculosis drug therapy, demonstrating the changes in the ascitic fluid’s characteristics and distribution. The improvement or alteration in the appearance of the fluid could indicate the therapeutic response and progression of the condition under treatment. In two patients (16.6%), the surfaces of the uterus, rectosigmoid colon, and small intestine showed thickening (Figure 4A).

**Figure 5:**
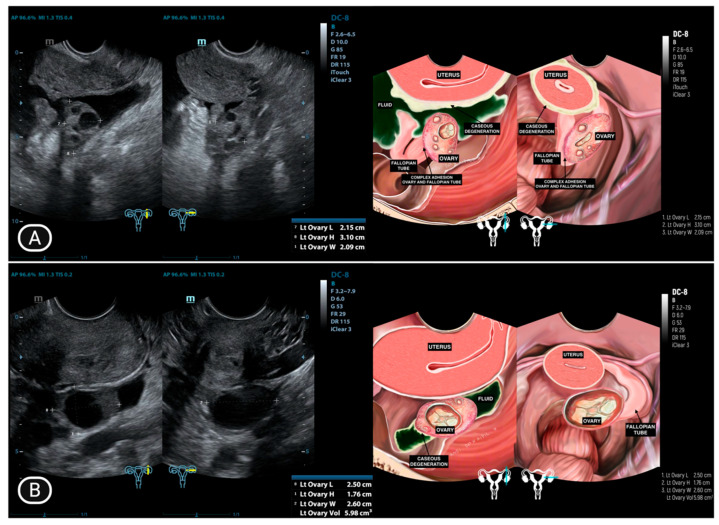
(**A**) Complex adhesion between the ovary and fallopian tube on the ipsilateral side, along with fluid accumulation in the peritoneal cavity; (**B**) Fluid and caseous degeneration decrease significantly in peritoneal cavity after one month of therapy with anti-tuberculosis drugs.

In 6 out of 12 patients (50%), complex adhesions were observed between the ovary and fallopian tube on the same side, as shown in Figure 5. These adhesions appeared on ultrasound as a hypoechoic, uniformly thickened structure measuring 4 to 8 mm, located beneath the anterior abdominal wall.

**Figure 6:**
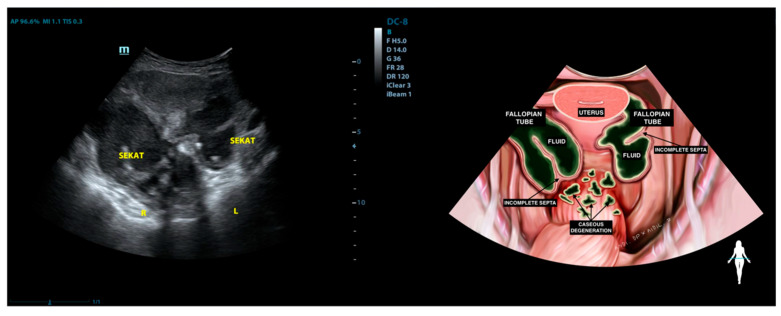
Formation of incomplete septa filled with complex fluid.

In seven out of 12 patients (58.3%), a hydrosalpinx appearance was noted, characterized by a mass with incomplete septa, containing caseous degeneration or complex fluid, as shown in Figure 6.

**Figure 7:**
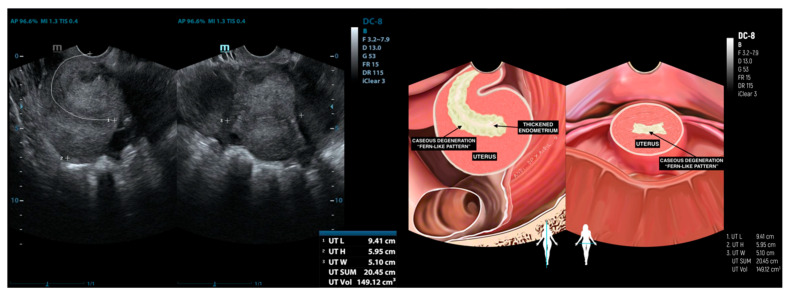
Accumulation of caseous degeneration in the endometrium characterized by a “fern-like pattern” and associated endometrial thickening.

In one patient (8.3%), the accumulation of caseous degeneration in the endometrium was observed, presenting a “fern-like pattern” and accompanied by thickening, as illustrated in Figure 7.

Table 3 presents biomarker data for 12 individuals, including CA-125, HE-4, CEA, ADA, and IGRA test. CA-125 levels are significantly elevated in most cases, with the highest recorded at 1425.8 U/mL. HE4 levels vary, with the highest at 130.4 pmol/L. Most individuals have low CEA levels, except in 1 case with 7.1 ng/mL, which could indicate another type of malignancy. IGRA tests show 9 out of 12 individuals tested positive, while 1 had an indeterminate result, and 1 case where a second test converted from negative to positive after one month. ADA levels are above 40 U/L in most cases, with the highest at 55 U/L.

The mean ± SD (Range) CA-125 level was 484.25 ± 357.8 (96.9–1425.8) U/mL, with all patients (100%) surpassing the normal reference value of ≤35 U/mL, suggesting significant elevation. The mean HE4 level was 66.8 ± 23.35 (47.7–130.4) pmol/L, with 5 patients (41.6%) exceeding the normal upper limit of ≤70 pmol/L. CEA showed a mean of 1.725 ± 1.727 (0.6–7.1) ng/mL, with only 1 patient (8.3%) exceeding the normal threshold of ≤5 ng/mL, reflecting minimal elevation. ADA had a mean of 45.8 ± 5.75 (39–55) U/L, with all patients surpassing the normal value of <30 U/L, consistent with an elevated response. IGRA results were positive in 9 patients (75%), supporting its utility as a diagnostic marker for peritoneal tuberculosis.

## 4. Discussion

In this study, the ultrasonography imaging revealed high rates of chronic pelvic infections, with hydrosalpinx in 7 cases (58.3%), often accompanied by adhesions and caseous degeneration. Adhesion complexes were present in 6 cases (50%), and ascites in 7 cases (58.3%). Rectosigmoid colon and small bowel involvement was seen in 2 (16.6%) cases, and cystic or mass-like abnormalities in 4 (33.3%) cases. Additionally, CA-125 and ADA were elevated in 12 (100%) cases, indicating significant deviations often linked to ovarian cancer and peritoneal tuberculosis. HE4 exceeded normal levels in 5 (41.6%) cases, and CEA was elevated in 1 (8.3%) case. IGRA showed a 75% positivity rate, reflecting a high prevalence of infections such as tuberculosis.

### 4.1. Ultrasonography Findings

The predominant ultrasound findings in individuals with PTB are ascites, and without meticulous sonographic evaluation, PTB may be erroneously identified as ovarian cancer [18]. Ascites refers to the abnormal accumulation of fluid, and in females, up to 20 cc in the pouch of Douglas is considered typical, contingent upon the period of the menstrual cycle [19]. Ascitic fluid is classified into two types: transudate (simple) and exudate (complex). Transudate appears anechoic on ultrasound, indicating it is free from solid particles or debris. In contrast, exudate shows debris, internal septa, and low-level echoes, suggesting the presence of proteins, cells, or microorganisms commonly associated with inflammation or infection [20]. In this study, US examination revealed ascites in 7 (58.3%) patients. Among them, 2 patients (16.67%) had free ascites, characterized by clear fluid accumulation. Two patients (16.67%) displayed complex ascites with low-level echoes, suggesting more complicated fluid buildup, possibly due to infection or malignancy. Another patient (8.3%) had fluid in the Douglas pouch with caseous degeneration, indicating potential inflammation. One patient (8.3%) was found to have a pocket abscess, a localized collection of pus. Lastly, one patient (8.3%) had massive ascites, where the fluid accumulation was extensive, causing significant abdominal distension. These ultrasonographic findings are in line with previous research, which has reported that complex ascites typically presents with similar features [4,21].

Half of the patients also exhibited complex adhesions between the ovary and fallopian tube on the same side, visible on ultrasound as a uniformly thickened hypoechoic structure. The hypoechoic appearance typically indicates fibrotic and inflammatory changes, which are characteristic of adhesive processes caused by infection or inflammation. The detection of such adhesions during ultrasound raises significant suspicion for tuberculosis infection, as this condition is often associated with the formation of fibrous bands or adhesions in the pelvic region, which can lead to fertility complications such as tubal obstruction or damage [22].

A hydrosalpinx appearance was observed in more than half of the patients, characterized by a mass with incomplete septa containing caseous degeneration or complex fluid. The longitudinal folds of the fallopian tube can thicken and become more noticeable on imaging, taking on a pattern similar to the teeth of a cogwheel. This gives rise to the distinctive “cogwheel” sign of hydrosalpinx when the tube is observed in cross-sectional view [23]. These findings are considered pathognomonic, meaning they are highly specific and indicative of a fluid-filled mass originating from the fallopian tube. Although such findings are commonly associated with tubo-ovarian abscesses (TOA), Oge et al. have reported that patients with pelvic tuberculosis who undergo laparotomy also display hydrosalpinx with caseous material and tubo-ovarian masses [24]. This correlation further confirms hydrosalpinx as a key feature of PTB-related pelvic pathology.

Thickening of the uterus, rectosigmoid colon, and small intestine surfaces was noted in two patients, possibly due to caseous degeneration. A comparable case described a fixed uterus, obliterated Douglas pouch peritoneum, and thickening of the meso-sigmoid-rectum, with histopathological examination confirming granulomatous lesions and caseous necrosis [24]. In another comparable case, tuberculosis was suspected during laparotomy due to the presence of pyosalpinx, a tubo-ovarian mass, and miliary tubercles, which were later confirmed by histopathology revealing caseous necrosis and Langhans giant cells [25].

The accumulation of caseous degeneration in the endometrium was observed in one patient. Endometrial thickening is a well-documented feature in genital tuberculosis affecting the endometrium, as reported in previous case studies [26]. Several studies indicate that the endometrium is the second most commonly involved site in genital TB, following the fallopian tubes [27]. Tuberculosis bacilli are primarily disseminated hematogenously, with the fallopian tubes being the initial site of infection, leading to endosalpingitis, followed by involvement of the endometrium. The infection may be retained in the basal layer or reintroduced after menstruation, resulting in the continuous formation of new tubercles. Additionally, retrograde dissemination to the ovaries and peritoneum may occur [28].

The sensitivity and specificity of ultrasonography for diagnosing PTB vary depending on the reference standard applied. A Cochrane systematic review published in 2019 evaluated the diagnostic accuracy of abdominal ultrasound for detecting abdominal tuberculosis or disseminated TB with abdominal involvement, particularly in HIV-positive individuals. This review reported a pooled sensitivity of 63% (95% CI: 43–79%) and a specificity of 68% (95% CI: 42–87%) when using bacteriological confirmation as the reference standard. These values increased to 68% (95% CI 45% to 85%) and 73% (95% CI 41% to 91%), respectively, when clinical diagnosis was used as the reference standard. Clinical diagnosis typically includes a combination of imaging findings, biomarker profiles, clinical presentation, and response to treatment [29]. This variation highlights the inherent challenges in diagnosing PTB, as microbiological confirmation is often difficult due to the paucibacillary nature of the disease. As a result, many true cases of PTB are diagnosed based on clinical and radiological evidence rather than definitive bacteriological proof. In the present study, a similar approach was adopted, integrating ultrasonographic features with biomarker data and clinical evaluation to support the diagnosis of PTB. This reflects current clinical practice, particularly in settings where reliance solely on microbiological confirmation is not always feasible or practical. The review also noted that ultrasound examinations are subjective and highly dependent on the operator, suggesting that subtle disease features may be overlooked in less experienced hands. Therefore, standardized training programs, adherence to diagnostic protocols, and, where possible, double-reading of ultrasound findings may improve diagnostic consistency and reliability.

### 4.2. Biomarker Profiles

This study evaluated a panel of biomarkers to support the diagnosis of PTB, with particular emphasis on their utility in distinguishing PTB from malignancies that present with overlapping clinical and radiological characteristics. The CA-125 antigen, widely recognized as a biomarker for ovarian cancer, is also elevated in various forms of tuberculosis, including PTB. This elevation is attributed to inflammation of the peritoneal lining, which stimulates CA-125 production. As a glycoprotein expressed on epithelial ovarian neoplasms and cells lining the endometrium, fallopian tubes, pleura, pericardium, and peritoneum, CA-125 increases in response to peritoneal irritation [13,30]. In this study, CA-125 levels were found to be elevated, with a mean concentration of 484.25 U/mL, ranging from 96.9 U/mL to 1425.8 U/mL, exceeding the normal reference level of <35 U/mL. These findings align with the study conducted by Zhang et al., which suggests that a CA-125 threshold of 563.5 U/mL may help differentiate PTB from ovarian cancer, with levels above this indicating ovarian cancer and levels below suggesting PTB [14]. However, given the wide array of conditions that can lead to elevated CA-125 levels, it cannot be considered a definitive diagnostic marker in isolation.

HE4 is a glycoprotein that functions as a proteinase inhibitor and is notably elevated in ovarian malignancies, particularly serous adenocarcinoma and endometrioid carcinoma. Unlike CA-125, which can rise in various benign conditions, HE4 offers greater specificity for malignancy, as its levels are generally unaffected by peritoneal inflammation. The combined use of CA-125 and HE4 has been shown to improve the differentiation between benign and malignant conditions [31,32], and PTB should be considered when CA-125 is elevated but HE4 remains within normal limits. In this study, the mean HE4 level was 66.8 pmol/L, ranging from 47.7 to 130.4 pmol/L, consistent with prior studies suggesting lower HE4 levels in PTB compared to ovarian cancer. Zhang et al. proposed a threshold of 151.4 pmol/L [14], while Angioli et al. suggested 262 pmol/L [33], both above the levels observed in our cohort. Although five patients had HE4 values exceeding 70 pmol/L, potentially due to renal dysfunction or comorbidities [34], these remained well below typical levels seen in malignancies.

Carcinoembryonic antigen (CEA) is a glycoprotein frequently elevated in adenocarcinomas of the gastrointestinal tract, breast, and lungs [35], with up to 90% of colorectal cancers showing elevated levels [36], and a reported specificity of 89% for early detection [37]. In PTB, where ascites is often exudative, elevated CA-125 may mimic carcinoma peritonitis. In such cases, CEA measurement aids in differentiation. Kaya et al. found that patients with exudative ascites, fever (>38 °C), elevated CA-125, and normal CA 19-9 and CEA levels, the specificity and positive predictive value for diagnosing PTB were both 100% [15]. In this study, CEA levels were measured to exclude colorectal malignancy. All patients showed low values, with a mean of 1.725 ng/mL, supporting the exclusion of gastrointestinal cancer and reinforcing PTB as the likely diagnosis.

Adenosine deaminase (ADA) is an enzyme involved in purine metabolism and T lymphocyte function. Its activity increases significantly in chronic inflammatory conditions, particularly in effusions such as pleural, peritoneal, and cerebrospinal fluid, often in response to tuberculosis [38,39]. In this study, ADA was measured in 7 patients with available ascitic fluid. All had levels exceeding the normal threshold (<30 U/L), with a mean of 45.8 U/L. These findings align with prior studies demonstrating high sensitivity and specificity of ADA in diagnosing PTB, with suggested cut-off values ranging from 39 to 40 U/L [16,40]. Furthermore, other studies have emphasized that ADA levels in ascites caused by TB infection are significantly higher compared to those in non-TB ascites, reinforcing the role of ADA as a reliable biomarker for distinguishing between TB-related and non-TB-related ascites [41].

Interferon-gamma release assays (IGRAs) are in vitro tests that measure Th1 cell responses to *Mycobacterium tuberculosis* antigens by detecting interferon-gamma (IFN-γ) release [42]. Su et al. reported a sensitivity of 93% and specificity of 99% for pulmonary TB detection using IGRA [17]. However, negative results alone do not exclude active TB, particularly in clinically suspected cases. In this study, 9 of 12 patients had positive IGRA results. One was initially negative but converted to positive after one month, and one yielded an indeterminate result. As IGRA typically becomes positive 2–12 weeks after exposure, delayed seroconversion is not uncommon [43]. Current guidelines recommend repeating IGRA testing after an initial negative result, especially if the patient had recent contact with a confirmed TB case or was tested within 8 weeks of exposure. Negative results obtained before 8 weeks should typically be confirmed by repeat testing 8–10 weeks after the exposure [44]. This practice ensures more accurate diagnosis by accounting for the possibility of a delayed immune response, as the immune system may take several weeks to mount a detectable response to Mtb infection.

This study is subject to several limitations. As a retrospective case series with a limited sample size, the generalizability of the findings may be constrained. The heterogeneity in diagnostic confirmation methods reflects the complexities of clinical practice, wherein some patients undergo surgical exploration while others are diagnosed based on a combination of imaging findings, biomarker profiles, and clinical assessment. It should be acknowledged that not all cases were confirmed through histopathological examination, which remains the diagnostic gold standard for peritoneal tuberculosis. The absence of uniform pathological confirmation across all subjects introduces a degree of diagnostic uncertainty that may influence the interpretation of results. Furthermore, although biomarkers such as CA-125, HE4, and CEA were incorporated to provide a more comprehensive clinical context, their limited specificity to peritoneal tuberculosis necessitates cautious interpretation. Additionally, ultrasonography is inherently operator dependent, which may result in interobserver variability in image acquisition and interpretation. Despite these limitations, the study contributes valuable insights into the ultrasonographic and biomarker characteristics associated with peritoneal tuberculosis and may inform diagnostic approaches, particularly in settings with limited access to definitive diagnostic modalities.

## 5. Conclusions

The diagnosis of PTB involves a comprehensive approach that combines clinical symptoms, ultrasound imaging, and biomarker measurements to ensure accurate identification. Clinically, PTB presents with lower abdominal pain, abdominal distension, intermittent low-grade fever, and irregular menstrual cycles. Ultrasound imaging plays a critical role in diagnosing PTB, as it often reveals hallmark features such as ascites, hydrosalpinx with caseous degeneration, caseous lesions on organ surfaces, and complex adhesions between the fallopian tube and ovary. These findings, however, can sometimes lead to misdiagnosis, with PTB being confused with other conditions such as Tubo-Ovarian Abscess (TOA) or even ovarian cancer, due to similar imaging characteristics. To complement these clinical and imaging findings, biomarkers such as CA-125, HE4, CEA, IGRA, and ADA are essential for diagnosing PTB.

In patients with PTB, CA-125 levels are typically elevated, whereas HE4 and CEA levels may remain normal or low. A positive result for IGRA and ADA levels in ascitic fluid further supports the diagnosis. In contrast, ovarian cancer, which can present with similar symptoms, results in significantly higher levels of CA-125 and HE4, and non-TB infections may show increased CA-125 levels but with normal or low HE4, along with negative IGRA and ADA results. By utilizing a combination of these diagnostic methods, healthcare providers can more accurately diagnose PTB and differentiate it from conditions such as TOA or ovarian cancer, reducing the need for more invasive procedures like laparoscopy or minilaparotomy. This approach not only enhances diagnostic accuracy but also allows for more timely and appropriate treatment, avoiding unnecessary invasive interventions.

## Figures and Tables

**Table 1:** Characteristics of research subjects (*n* = 12).

Characteristics	Value
Mean age (±SD)	33.0 ± 9.7 years
Median age (range)	30.5 (16–53) years
Menstrual Status	
Premenopausal	10 (83.3%)
Postmenopausal	2 (16.7%)
Marital Status	
Married	9 (75%)
Not Married	3 (25%)
History of TB Exposure	
Yes	3 (25%)
No/Unknown	9 (75%)
Histopathology-confirmed TB	
Yes	4 (33.3%)
No/Clinical Diagnosis only	8 (66.7%)

**Table 2:** Ultrasonographic Findings of Study Participants (*n* = 12).

No.	Initial (Age)	Uterus	Right Ovarium	Right Fallopian Tube	Left Ovarium	Left Fallopian Tube	Ascites	Other Organ Surfaces
1	LA (25)	Normal	Adhesion complex with hydrosalpinx right fallopian tube	Hydrosalpinx, filled with caseous degeneration inside	Adhesion complex with hydrosalpinx left fallopian tube	Hydrosalpinx, filled with complex fluid	Complex ascites	(−)
2	AY (33)	Normal	Normal	Normal	Normal	Occlusion	Free ascites	(−)
3	IO (31) *	Caseous degeneration on the surface	Adhesion complex	Normal	Adhesion complex	Normal	(−)	Caseous degeneration on the surface of rectosigmoid colon and small bowel
4	SMK (29) *	Caseous degeneration on the surface. Complex fluid behind uterus	Normal	Not visualized	Adhesion complex with left fallopian tube	Hydrosalpinx	Complex fluid in cavum of Douglas with caseous degeneration	Caseous degeneration on the surface of rectosigmoid colon
5	TDW (45)	Normal	Normal	Not visualized	Normal	Normal	Free ascites	(−)
6	STG (53)	Adherent to both masses in the adnexa and the rectosigmoid	Not visualized	Mass with incomplete septation (Hydrosalpinx)	Normal	Mass with incomplete septation (Hydrosalpinx)	(−)	(−)
7	EF (30)	Normal	Normal	Normal	Normal	Mass with incomplete septation, filled with complex fluid (Hydrosalpinx)	(−)	(−)
8	OO (16)	Normal	Normal	Not visualized	Normal	Not visualized	Pocket Abscess	(−)
9	IHS (44)	Normal	Enlarged, cystic, unilocular, and contains simple fluid	Not visualized	Normal	Not visualized	Massive ascites	(−)
10	AS (44) *	Ill−defined mass at the anterior	Adhesion complex with hydrosalpinx right fallopian tube	Hydrosalpinx	Enlarged with multilocular cystic filled with complex fluid	Not visualized	(−)	(−)
11	Y (30) *	Normal	Adhesion to pelvic wall	Not visualized	Adhesion complex with left fallopian tube	Hydrosalpinx, filled with caseous degeneration inside	Complex ascites	(−)
12	DR (24)	Complex fluid in uterine cavity (10.98 mm)	Normal	Hydrosalpinx	Adhesion	Normal	(−)	(−)

* Histopathology-confirmed peritoneal tuberculosis. The”−” symbol signifies its absence.

**Table 3:** Patient-Specific Biomarker Data for Peritoneal Tuberculosis Diagnosis.

No.	Initial (Age)	CA-125 (U/mL)	HE-4 (pmol/L)	CEA (ng/mL)	ADA (U/L)	IGRA
1	LA (25)	612.8	79.4	1.5	49	1st test: Negative 2nd test: Positive (Repeated after one month)
2	AY (33)	836.3	47.7	0.6	50	Positive
3	IO (31) *	337.7	73.3	1.2	-	Positive
4	SMK (29) *	460.4	77.0	0.9	44	Positive
5	TDW (45)	333	47.8	1.1	40	Positive
6	STG (53)	358.8	50.9	1.3	-	Positive
7	EF (30)	1425.8	50.6	1.2	-	Positive
8	OO (16)	183.1	52.2	1.1	55	Indeterminate
9	IHS (44)	208.8	60.0	1.9	44	Positive
10	AS (44) *	525.2	74.7	1.7	-	Positive
11	Y (30) *	432.2	130.4	7.1	39	Positive
12	DR (24)	96.9	57.6	1.1	-	Negative

* Histopathology-confirmed peritoneal tuberculosis.

## Data Availability

The data that support the findings of this study are available from the corresponding author upon reasonable request.
